# Disruption of the NF-κB/IκBα Autoinhibitory Loop Improves Cognitive Performance and Promotes Hyperexcitability of Hippocampal Neurons

**DOI:** 10.1186/1750-1326-6-42

**Published:** 2011-06-10

**Authors:** David J Shim, Li Yang, J Graham Reed, Jeffrey L Noebels, Paul J Chiao, Hui Zheng

**Affiliations:** 1 Huffington Center on Aging, Baylor College of Medicine, Houston, TX 77030, USA; 2 Department of Neuroscience, Baylor College of Medicine, Houston, TX 77030, USA; 3 Medical Scientist Training Program, Baylor College of Medicine, Houston, TX 77030, USA; 4 Department of Molecular and Human Genetics, Baylor College of Medicine, Houston, TX 77030, USA; 5 Department of Neurology, Baylor College of Medicine, Houston, TX 77030, USA; 6 Department of Surgical Oncology-Research, University of Texas M. D. Anderson Cancer Center, Houston, TX 77030, USA

## Abstract

**Background:**

Though originally discovered in the immune system as an important mediator of inflammation, NF-κB has recently been shown to play key roles in the central nervous system, such as synaptogenesis, synaptic plasticity, and cognition. NF-κB activity is normally tightly regulated by its primary inhibitor, IκBα, through a unique autoinhibitory loop. In this study, we tested the hypothesis that the IκBα autoinhibitory loop ensures optimal levels of NF-κB activity to promote proper brain development and function. To do so, we utilized knock-in mice which possess mutations in the IκBα promoter to disrupt the autoinhibitory loop (IκBα^M/M ^KI mice).

**Results:**

Here, we show that these mutations delay IκBα resynthesis and enhance NF-κB activation in neurons following acute activating stimuli. This leads to improved cognitive ability on tests of hippocampal-dependent learning and memory but no change in hippocampal synaptic plasticity. Instead, hippocampal neurons from IκBα^M/M ^KI mice form more excitatory and less inhibitory synapses in dissociated cultures and are hyperexcitable. This leads to increased burst firing of action potentials and the development of abnormal hypersynchronous discharges *in vivo*.

**Conclusions:**

These results demonstrate that the IκBα autoinhibitory loop is critical for titrating appropriate levels of endogenous NF-κB activity to maintain proper neuronal function.

## Background

The transcription factor NF-κB, originally discovered in B cells of the immune system [1], has since been shown to be expressed in nearly all cell types. Beyond its role as a critical regulator of the inflammatory response, NF-κB activation can also promote the expression of genes involved in apoptosis and cell survival, thus making it an important mediator of the general stress response [2]. In the central nervous system (CNS), NF-κB signaling regulates neuronal survival following acute pathologic damage such as traumatic brain injury [3-5] and stroke [6,7] as well as in chronic neurodegenerative diseases such as Alzheimer's disease [8,9] and Parkinson's disease [10,11]. NF-κB also plays an important role in normal development and function of the brain. Recent studies have shown that NF-κB activation is required during long term memory formation [12-16] as well as during induction of synaptic plasticity [14,16-18]. Furthermore, NF-κB plays an important role in neurite outgrowth and synaptogenesis [19-21]. Indeed, NF-κB is present in post-synaptic compartments and is rapidly transported to the nucleus following stimulation with glutamate or depolarization with KCl [13].

NF-κB is normally bound by an inhibitory IκB protein and sequestered in the cytoplasm. Activation of NF-κB classically requires the phosphorylation, ubiquitination, and proteasome-mediated degradation of the IκB protein, thereby freeing NF-κB to translocate to the nucleus [22,23]. NF-κB is quickly silenced by the rapid resynthesis of its principal inhibitor, IκBα [24]. This occurs via direct binding of NF-κB to consensus κB sites in the promoter for IκBα, thus forming a powerful mechanism of feedback inhibition [25]. Complete knockout of IκBα leads to early postnatal lethality in mice [26,27], highlighting the vital importance of IκBα-mediated inhibition of NF-κB. To investigate the role of the IκBα autoinhibitory loop on NF-κB signaling, we have generated knock-in mice possessing mutations of the κB sites in the IκBα promoter (IκBα^M/M ^KI mice), thus specifically abrogating the NF-κB-mediated feedback arm of this autoinhibitory loop [28]. Here, we show that genetic disruption of this loop leads to enhanced NF-κB activity in neurons. While IκBα^M/M ^KI mice display increased cognitive ability on hippocampal-dependent behavioral tasks, hippocampal plasticity is unchanged. Instead, increased NF-κB signaling alters the balance of excitatory and inhibitory synaptogenesis, leading to hyperexcitability and increased spontaneous burst firing in hippocampal cultures and acute slices. As a consequence, IκBα^M/M ^KI mice display increased seizure-like activity. Together, these data reveal the importance of tight regulation of NF-κB signaling for proper neuronal function.

## Methods

### Mouse breeding

Mice were housed 2-5 per cage with *ad libitum *access to food and water in a room with a 12 h light/dark cycle in a sterile pathogen-free mouse facility. All procedures were performed in accordance with NIH guidelines and with the approval of the Baylor College of Medicine Institutional Animal Care and Use Committee. Initial IκBα^M/M ^KI mice were generated as described previously [28]. For the current studies, mice were further backcrossed onto a pure C57BL/6 background for a minimum of five generations. Heterozygous mice were then intercrossed to obtain littermate IκBα^+/+ ^(WT) mice or IκBα^M/M ^KI mice. For our behavioral experiments, we subsequently set up WT × WT and KI × KI breeding cages to obtain larger cohorts of age-matched male mice. Genotyping was performed by PCR of tail DNA at time of weaning.

### Neuronal culture

Neonatal pups were collected from heterozygous breeding cages and genotyped. For molecular experiments, whole brains were removed and pooled by genotype into ice cold dissection buffer (Hanks Buffered Saline Solution supplemented with 10 mM HEPES, pH 7.5, 0.6% glucose, 20 U/ml penicillin, 20 μg/ml streptomycin). Using a dissecting microscope, cortices were isolated and meninges removed. The tissue was then cut into small pieces and transferred to 10 ml of dissection buffer. Following addition of 500 μl trypsin (2.5%), the tissue was incubated at 37°C for 12 min. 400 μl soybean trypsin inhibitor (1 mg/ml) and 100 μl DNase I (1%) were then added and the tissue collected by centrifugation for 5 min at 1200 rpm. The supernatant was decanted off and replaced with 2 ml of culture media (Neurobasal medium supplemented with 2% B27, 0.5 mM L-glutamine, 40 U/ml penicillin, 40 μg/ml streptomycin) and 20 μl DNase I (1%). The tissue was then gently triturated with a cut P1000 pipette tip 8-10 times. After allowing the remaining pieces to settle, the supernatant was collected into a fresh tube and the remaining tissue pieces were again triturated in 2 ml fresh culture media with an uncut P1000 pipette tip. This was repeated once more, after which no tissue pieces were visible. The collected supernatants were then centrifuged for 5 min at 1200 rpm and the cell pellet resuspended in 5 ml culture media. This was repeated once more before the cells were counted and plated onto poly-D-lysine coated 6 cm dishes at a density of 2.5-3 × 10^6 ^cells/dish in a volume of 5 ml culture media. For electrophysiology and immunostaining experiments, only hippocampi were isolated and plated onto poly-D-lysine coated glass coverslips in 24-well plates at a density of 1 × 10^5 ^cells/well. To improve local density, cells were initially applied to the center of the coverslip in a 40 μl bubble and allowed to settle before filling the well with 500 μl culture media. All cultures were grown in tissue culture incubators at 37°C, 5% CO_2_, 95% humidity.

### Protein isolation and Western blot

To measure NF-κB kinetics, cortical cultures were stimulated with 10 ng/ml recombinant TNFα (Chemicon, GF023) or IL-1β (Calbiochem, 407617) at 10 DIV by replacing half the culture media with fresh culture media containing 2x drug for the indicated duration. The media was then completely removed and the neurons were scraped into 100 μl Buffer A (10 mM HEPES, pH 7.9, 1.5 mM MgCl_2_, 10 mM KCl, 500 μM DTT) supplemented with complete protease and phosphatase inhibitor cocktails. The lysate was incubated on ice for 1 hr followed by addition of 5 μl 10% NP-40 and then vortexed for 10 sec. The lysate was then centrifuged for 20 sec at 14,000 rpm and the supernatant containing cytoplasmic proteins was collected and stored at -80°C for future use. The pellet was then incubated in 50 μl Buffer C (20 mM HEPES, pH 7.9, 25% glycerol, 420 mM NaCl, 1.5 mM MgCl_2_, 0.2 mM EDTA, 500 μM DTT) supplemented with complete protease and phosphatase inhibitor cocktails and vortexed for 10 sec every 10-15 min for a total of 1 hr. The resulting lysate was then spun at 4°C for 5 min at 14,000 rpm and the supernatant containing nuclear proteins was collected and stored at -80°C for future use. For Western blot analysis, the cytoplasmic fractions were quantified using a DC colorimetric protein assay (Bio-Rad) and boiled at 95°C for 7 min in sample buffer. 15 μg of protein samples were then loaded onto 12% SDS-polyacrylamide gels, run at 100 mV for 2 hr, transferred onto nitrocellulose membranes (Bio-Rad) at 90 V for 1.5 hr at 4°C in transfer buffer (50 mM Tris, 40 mM glycine, 20% methanol, 0.01% SDS), and then blocked with 5% milk in Tris-buffered saline containing 0.1% Tween-20 (TBST). The membranes were then probed with primary antibody (Rabbit anti-IκBα, Santa Cruz, sc-371, 1:1000; Mouse anti-α-tubulin, Sigma, 1:20,000) diluted in blocking solution overnight at 4°C. Membranes were washed 4 × 10 min in TBST and then blotted with secondary antibody (Horse anti-Rabbit-HRP, Vector Labs, 1:5000; Horse anti-Mouse-HRP, Vector Labs, 1:5000) for 2 hr at room temperature. The membranes were again washed 4 × 10 min in TBST, incubated in ECL solution (GE Healthcare Life Sciences), and exposed to film. After developing, the films were digitized on a flatbed scanner and band intensities quantified using ImageJ software (NIH). Levels of α-tubulin served as control for loading.

### EMSA and NF-κB p65 ELISA

For EMSA experiments, NF-κB consensus probes (Santa Cruz, sc-2505) were end-labeled with ^32^P using T4 polynucleotide kinase (New England Biolabs) and purified using nucleotide purification columns (Qiagen). 1 μl of labeled probe was then mixed with 1 μl poly dI:dC, 3 μl 5x binding buffer (75 mM Tris, pH 7.5, 375 mM NaCl, 7.5 mM EDTA, 25% glycerol, 100 μg/ml BSA), and 3-5 μg of nuclear protein in a final volume of 15 μl. For cold competition, 1 μl of unlabeled consensus or mutant (Santa Cruz, sc-2511) probe was added at 10-fold excess. For supershift, 1 μl of anti-p50 (Santa Cruz, sc-1190X), or anti-p65 (Santa Cruz, sc-372X) antibody was added to the mixture. After a 30 min incubation at 4°C, the samples were loaded onto a 6% non-denaturing polyacrylamide gel and run in 0.5x TBE at 4°C for 30 min at 200 V, then 2 hr at 250 V. The gel was then dried down onto filter paper using a slab gel dryer and then exposed by autoradiography to Kodak-MR film at -80°C. For quantification of p65, nuclear samples were analyzed with the ELISA-based TransAM NFκB p65 kit (Active Motif) according to the manufacturer's instructions.

### qRT-PCR

For quantitative real-time PCR experiments, total RNA was isolated from cortical neuronal cultures and analyzed as described previously [29]. The primer sequences are as follows: 5'-TCGCTCTTGTTGAAATGTGG-3' (IκBα-Fwd), 5'-TCATAGGGCAGCTCATCCTC-3' (IκBα-Rev), 5'-AATGTGTCCGTCGTGGATCTGA-3' (GAPDH-Fwd), and 5'-GATGCCTGCTTCACCACCTTCT-3' (GAPDH-Rev).

### Behavioral assays

All mouse behavior experiments were performed in the Mouse Neurobehavior Core facility with age-matched cohorts of 2-4 mo old male mice from homozygous WT or KI breeding. The initial test battery consisted of, in order, open field, light-dark, rotarod, prepulse inhibition, conditioned fear, and hotplate. There was a minimum separation of one day between tests, which has been shown previously not to have significant carryover effect using the same test protocols and equipment [30,31]. Additional cohorts of mice were later tested with elevated plus maze or Morris water maze as described previously [32].

### Electrophysiology

For extracellular field potential recordings, brains were isolated from 3-5 mo old mice and cut into 400 μM horizontal or transverse slices using a vibratome sectioning system (PELCO) in ice cold cutting artificial cerebral spinal fluid (cutting ACSF: 110 mM sucrose, 60 mM NaCl, 3 mM KCl, 1.25 mM NaH_2_PO_4_, 28 mM NaHCO_3_, 7 mM MgCl_2_, 0.5 mM CaCl_2_, 5 mM glucose, and 0.6 mM ascorbate, saturated with 95% O_2 _and 5% CO_2_). Hippocampal slices were then transferred to a heated recording chamber filled with recording ACSF (125 mM NaCl, 2.5 mM KCl, 1.25 mM NaH_2_PO_4_, 25 mM NaHCO_3_, 1 mM MgCl_2_, 2 mM CaCl_2_, and 10 mM glucose, saturated with 95% O_2 _and 5% CO_2_) maintained at 32°C. Stimulation of Schaffer collaterals from the CA3 region was performed with bipolar electrodes, while borosilicate glass capillary pipettes filled with recording ACSF (resistances of 1 to 1.5 MΩ) were used to record field excitatory postsynaptic potentials (fEPSPs) in the CA1 region. Signals were amplified using a MultiClamp 700 B amplifier (Axon), digitized using a Digidata 1322A (Axon) with a 3 kHz low pass filter and a 0.1 Hz high pass filter, and then captured and stored using Clampex 9 software (Axon) for offline data analysis. Before each experiment, input-output recordings were made by stimulating for 0.1 ms at intensities ranging from 50 to 500 μA and calculating the slope of the fEPSP responses. A stimulation intensity corresponding to 30% of the maximal fEPSP slope was then chosen. For paired-pulse experiments, pairs of stimuli were delivered with interpulse intervals ranging from 10 to 200 ms and the fEPSP amplitude from the second stimulus (P2) was divided by the fEPSP amplitude from the first stimulus (P1) to calculate paired pulse facilitation (P2/P1). For LTP measurement, baseline transmission was measured every 20 sec for 10 min. A weak LTP induction protocol was then applied using one train of theta burst stimulation (1 × TBS) consisting of ten 5 Hz clusters of four 100 Hz pulses. fEPSP traces were again recorded for 20 min before a standard LTP induction protocol was applied using three trains of TBS (3 × TBS) spaced 20 sec apart, followed by 60 min of fEPSP recording. For data analysis, every 3 consecutive fEPSP traces were averaged together and the initial slope of the fEPSP measured. For spontaneous burst activity, the recording electrode was placed in either CA3 or CA1 and the perfusion buffer changed to modified ACSF containing 8.5 mM K^+ ^and 0.5 mM Mg^2+ ^as described previously [33]. Five consecutive traces of 50 sec each were recorded and the number of interictal bursts counted manually. For whole cell patch clamp experiments, hippocampal cultures were transferred to a recording chamber perfused with oxygenated Tyrode solution (25 mM HEPES, 129 mM NaCl, 5 mM KCl, 2 mM CaCl_2_, 1 mM MgCl_2_, 30 mM glucose, 10 μM glycine, and 50 μM picrotoxin). Neurons were recorded under whole cell patch using electrodes filled with recording solution (40 mM HEPES, pH 7.2, 110 mM K-Gluconic acid, 10 mM phosphocreatine, 10 mM EGTA, 2 mM MgATP, 2 mM Na_2_ATP, 0.3 mM Na_2_GTP) with a resistance of 3-8 MΩ. For spontaneous firing, neurons were held in current clamp mode without any current injection and recorded for 100 sec. For evoked firing, neurons were held in current clamp mode and steps of current ranging from 0 to 200 pA (in 20 pA increments) were injected for 100 ms and the number of action potentials elicited were counted.

### Immunostaining

To quantify synaptic puncta formation, 14-15 DIV hippocampal neuronal cultures were washed with phosphate buffered saline (PBS) 2 × 5 min and then fixed in 4% PFA overnight at 4°C. Coverslips were again washed with PBS 3 × 5 min before permeabalization in PBS with 0.1% Triton X-100 (PBST) for 15 min at room temperature. Neurons were then blocked with 3% goat serum in PBST for 1 hr and then stained with primary antibody (Rabbit anti-VGLUT1, Synaptic Systems, 1:2000; Mouse anti-MAP2, Chemicon, 1:2000; Mouse anti-VGAT, Synaptic Systems, 1:1000; Rabbit anti-MAP2, Chemicon, 1:1000; Mouse anti-PSD-95, Chemicon, 1:500; Rabbit anti-GAD65, Chemicon, 1:500; Mouse anti-Gephyrin, Synaptic Systems, 1:500) diluted in blocking solution overnight at 4°C. Coverslips were then washed in PBST 5 × 3 min before incubation with secondary antibody (Goat anti-Rabbit-Alexa555, Invitrogen, 1:2000; Goat anti-Mouse-Alexa488, Invitrogen, 1:2000) diluted in blocking solution for 2 hr at room temperature. Coverslips were again washed in PBST 5 × 3 min and then mounted onto glass slides with Prolong Gold AntiFade Reagent with DAPI (Invitrogen). The coverslips were sealed with clear nail polish and stored at 4°C. Images were taken using a 63x oil objective on a Nikon epifluorescent microscope using Metamorph software. Image analysis was performed using ImageJ software. For colocalization experiments, images were split into red and green channels and a threshold was applied. Presynaptic puncta were automatically outlined and the percent area which also stained positive for each postsynaptic marker was calculated. Puncta which contained at least 25% overlap were considered positive.

### Video-EEG recording

For analysis of seizure-like activity, mice were anaesthetized and cranial burr holes drilled to allow placement of electrodes. Teflon-coated silver wire electrodes (0.005 inch diameter) were implanted in the subdural space overlying temporal and parietal cortex bilaterally. The electrodes were then connected to a microminiature connector (Omnetics). After a 24 hr recovery period, simultaneous video and EEG recordings were captured in freely behaving mice for multiple 2-4 hr intervals over a one week period with a computer system running Harmonie software (Stellate Systems).

### Statistics

All data is presented as mean ± SEM. Outliers were identified using Grubbs' method with α = 0.05. Pairwise comparisons were analyzed using a two-tailed Student's *t*-test, while a two-way ANOVA followed by Bonferroni post-hoc analysis was used for multiple comparisons. P values less than or equal to 0.05 were considered statistically significant.

## Results

### Mutation of the IκBα promoter delays IκBα resynthesis and enhances NF-κB activity in neurons

**Figure 1:**
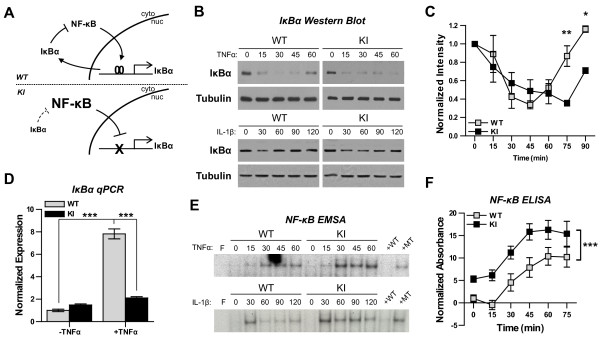
**Molecular characterization of NF-κB/IκBα dynamics in IκBα^M/M ^KI neurons**. **A) **Diagram depicting the IκBα autoinhibitory loop and the effect of our KI mutations. **B) **Representative Western blots of IκBα protein levels in cytoplasmic fractions from WT and KI primary neurons treated with TNFα (top) or IL-1β (bottom) for the indicated length of time. **C) **Quantification of Western blots of IκBα protein levels following TNFα stimulation normalized to the loading control and to baseline expression. N = 2-5 cultures each, * = p < 0.05, ** = p < 0.01. **D) **Expression of IκBα mRNA was measured by quantitative RT-PCR in WT and KI primary neurons before and after TNFα stimulation for 1 hr and levels were normalized to GAPDH expression and then to WT unstimulated levels. N = 3 cultures each, *** = p < 0.001. **E) **Representative EMSAs of NF-κB activity in nuclear fractions from WT and KI primary neurons treated with TNFα (top) or IL-1β (bottom) for the indicated length of time. Specificity of the shifted band is confirmed through the addition of either excess unlabeled consensus (WT) or mutant (MT) probe in the last two lanes. Free labeled probe (F) alone was run in the first lane. **F) **Quantification of NF-κB activity from nuclear fractions following TNFα stimulation using an ELISA-based assay specific for the p65 subunit. N = 3-4 cultures each, *** = p < 0.001.

Genetic disruption of the IκBα autoinhibitory loop (Figure 1A) leads to delayed resynthesis of IκBα and subsequent enhancement of NF-κB activity in peripheral tissues [28]. To address the role of IκBα autoinhibition in neurons, we stimulated primary neuronal cultures from WT or IκBα^M/M ^KI mice with the inflammatory cytokine TNFα (10 ng/ml) and measured the degradation and resynthesis of IκBα protein. In cultures from WT mice, TNFα stimulation led to the degradation of IκBα until, on average, 34% remained at 45 min. This was followed by a rise in IκBα protein levels, despite the continued presence of TNFα, until near normal levels of IκBα protein were detectable at 75 min and 90 min (Figure 1B, top, 1C). In contrast, neuronal cultures from IκBα^M/M ^KI mice stimulated with TNFα displayed degradation of IκBα which persisted through 75 min of treatment (Figure 1B, top, 1C, p < 0.01), and only began to rise after 90 min (Figure 1C, p < 0.05). Similar results were seen when we stimulated neuronal cultures with the cytokine IL-1β (Figure 1B, bottom). Hence, resynthesis of IκBα after stimulus-induced degradation is delayed in IκBα^M/M ^KI neuronal cultures compared to WT neuronal cultures. To determine whether the difference is due to a change in the transcription of IκBα, we examined the expression of IκBα using quantitative RT-PCR in WT and IκBα^M/M ^KI neuronal cultures stimulated with TNFα. Treatment for 1 hr led to a nearly 8-fold increase in IκBα mRNA levels in WT neurons (Figure 1D, p < 0.001), however this enhancement was significantly blunted in IκBα^M/M ^KI neurons (Figure 1D, p < 0.001). These results confirm that genetic mutation of the κB binding sites in the IκBα promoter leads to a loss in stimulus-induced expression of IκBα and delays the resynthesis of IκBα in cultured neurons. To assess the effect of delayed resynthesis of IκBα on NF-κB kinetics, we examined a time course of nuclear NF-κB binding activity after stimulation using electrophoretic mobility shift assay (EMSA). In WT neurons treated with TNFα, NF-κB binding activity was clearly detectable at 30 min and persisted through 60 min (Figure 1E, top). In IκBα^M/M ^KI neurons, TNFα stimulation also led to an increase in NF-κB activation but to a higher degree compared to WT neurons (Figure 1E, top). This enhancement in NF-κB activity was also seen following stimulation with IL-1β (Figure 1E, bottom). To quantify this difference in activation, we utilized an ELISA-based assay which captures NF-κB using immobilized DNA oligos and detects bound NF-κB with an antibody specific for the p65 subunit of NF-κB. The results of this experiment confirmed the higher level of NF-κB activation in KI neurons compared to WT neurons following stimulation with TNFα (Figure 1F, p < 0.05). Together, these results demonstrate that IκBα^M/M ^KI mutations disrupt the IκBα autoinhibitory loop in neurons by delaying resynthesis of IκBα, leading to an enhancement of NF-κB activity in response to acute activating stimuli.

### Hippocampal-dependent learning and memory is enhanced in IκBα^M/M ^KI mice

**Figure 2:**
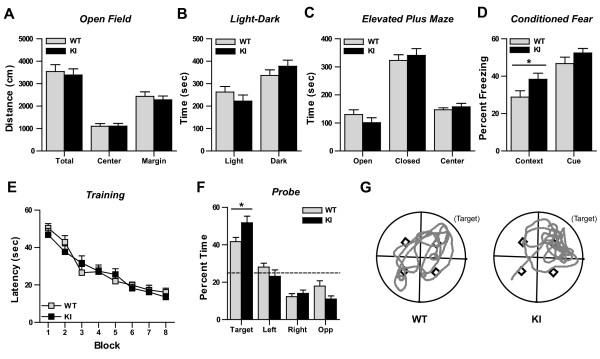
**Selective enhancement of hippocampal-dependent learning and memory in IκBα^M/M ^KI mice**. **A) **No change in activity levels on the open field assay. N = 13-14 mice. **B) **No change in anxiety-like behavior in light-dark assay. N = 13-14 mice. **C) **No change in anxiety-like behavior on the elevated plus maze. N = 13-14 mice. **D) **Increased freezing in response to context but normal freezing in response to cue following conditioned fear training. N = 12-21 mice, * = p < 0.05. **E) **No difference in latency to find the platform during the Morris water maze training phase. N = 17 mice. **F) **Both WT and KI mice display preference for the target quadrant during the Morris water maze probe test, however KI mice spend significantly more time in the target quadrant. Dashed line indicates chance performance. N = 17 mice, * = p < 0.05. **G) **Example traces showing swim path during the probe test. Black squares indicate possible platform locations in each of the quadrants.

Numerous studies have implicated a critical role for NF-κB in learning and memory processes [34,35]. We therefore asked whether blocking NF-κB- mediated autoinhibition alters learning and memory by assaying behavior in IκBα^M/M ^KI mice. First, we tested our mice with a battery of assays to determine whether general aspects of behavior are affected. We found no changes in activity levels using the open field assay (Figure 2A), and no evidence of increased anxiety-like behavior on either the light-dark assay (Figure 2B) or elevated plus maze (Figure 2C). Furthermore, we detected no difference in nociception using the hotplate assay (WT = 7.08 ± 0.65 sec vs. KI = 6.32 ± 0.50 sec, N = 14-20 mice, p = 0.36). These results demonstrate that general behaviors are largely intact in IκBα^M/M ^KI mice and allow the use of more complex behavioral paradigms to measure learning and memory. We next tested age-matched cohorts of 2-4 mo old WT and IκBα^M/M ^KI mice using the conditioned fear assay in which a mild footshock is paired with an auditory cue, and the mice are tested 24 hr later for their response to either the context in which they received the footshock or the cue that was paired with the footshock. IκBα^M/M ^KI mice demonstrated increased freezing to the context where they received the shock compared to WT mice (Figure 2D, p < 0.05). This was specific to the contextual test as WT and IκBα^M/M ^KI mice exhibited similar levels of freezing in response to the auditory cue alone (Figure 2D). Generally, both contextual and cued fear memory expression are thought to require proper amygdalar function, whereas the hippocampus is thought to be specifically required for contextual fear memory [36]. The selective increase in contextual freezing suggests that hippocampal-dependent learning and memory is improved in IκBα^M/M ^KI mice, perhaps due to enhanced NF-κB activity in hippocampal neurons. We next used the Morris water maze assay to test spatial memory, a second form of hippocampal-dependent learning and memory. Cohorts of age-matched 3-4 mo old WT and IκBα^M/M ^KI mice were trained with blocks of 4 swim trials, 2 blocks/day, over four days. Both groups learned to find the hidden platform at approximately the same rate during the training phase (Figure 2E). After training, mice were tested in a probe trial with the target platform removed, and the percent of time spent in the target quadrant where the platform had been present during training was measured. IκBα^M/M ^KI mice spent more time in the target quadrant compared to WT mice (Figure 2F-G, p < 0.05), indicating enhanced retention of the memory. These results demonstrate an improvement in spatial memory in IκBα^M/M ^KI mice, and together with the improved contextual fear memory, suggest that enhanced NF-κB signaling improves cognitive performance by altering hippocampal function.

### Synaptic plasticity is unchanged in IκBα^M/M ^KI hippocampal slices

**Figure 3:**
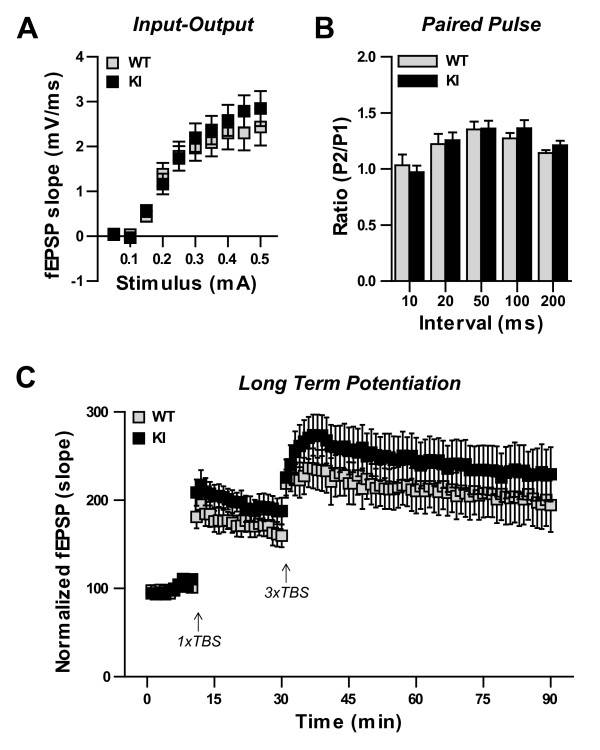
**Normal hippocampal synaptic plasticity in IκBα^M/M ^KI mice**. **A) **No change in input-output relationship. N = 15-17 slices. **B) **No change in paired pulse facilitation at the indicated inter-pulse intervals. N = 26-34 slices. **C) **Similar changes in fEPSP slope following a weak (1 × TBS) and standard (3 × TBS) LTP induction stimulus. N = 14-20 slices.

Synaptic plasticity has long been proposed as the cellular mechanism underlying behavioral learning and memory [37] and NF-κB has been shown to play a role in several forms of synaptic plasticity [14,16-18]. Given that our IκBα^M/M ^KI mice display enhancement of two separate forms of hippocampal-dependent learning and memory, we decided to test whether long term potentiation (LTP) was affected in the hippocampus using electrophysiology. We therefore prepared acute hippocampal slices from WT and IκBα^M/M ^KI mice and recorded extracellular field excitatory post-synaptic potentials (fEPSPs) at CA3-CA1 synapses. First, we tested baseline synaptic transmission by measuring the fEPSP in response to a range of stimulus currents and found no difference in the input-output relationship between WT and IκBα^M/M ^KI slices (Figure 3A). Next we tested paired pulse facilitation, a form of short term synaptic plasticity, and found no difference between WT and IκBα^M/M ^KI slices (Figure 3B). Finally, after a 10 min baseline recording, we induced LTP using a relatively weak protocol, one train of theta burst stimulation (1xTBS), and measured the resulting change in synaptic strength for 20 min. We then further induced LTP with a standard protocol, three trains of theta burst stimulation (3 × TBS), and again measured the change in synaptic strength for 60 min. Following both the weak and standard induction protocols, we saw similar increases in fEPSP slope in both WT and IκBα^M/M ^KI slices (Figure 3C). These results indicate that basal synaptic transmission and plasticity are normal in IκBα^M/M ^KI hippocampal networks and suggest that alternate mechanisms underlie the improved cognitive performance.

### Enhanced NF-κB activity alters the balance of excitatory to inhibitory synapse density

**Figure 4:**
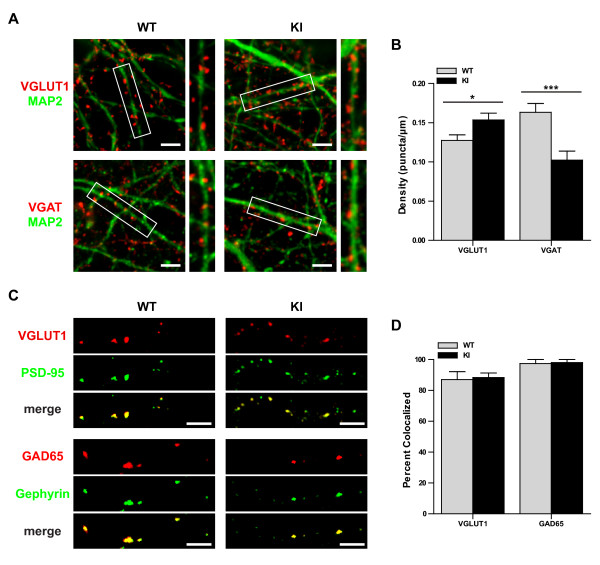
**Altered synaptogenesis in IκBα^M/M ^KI hippocampal neurons**. **A) **Representative images of hippocampal neuronal cultures from WT and KI mice stained with a dendritic marker (MAP2/green) and either an excitatory presynaptic marker (VGLUT1/red) or an inhibitory presynaptic marker (VGAT/red). Area in white box is shown in higher detail to the right. Scale bar = 5 μm. **B) **Quantification of VGLUT1 and VGAT puncta density. N = 37-61 segments, * = p < 0.05, *** = p < 0.001. **C) **Representative images showing colocalization of presynaptic and postsynaptic markers in excitatory synapses (VGLUT1/PSD-95) and inhibitory synapses (GAD65/Gephyrin). Scale bar = 5 μm. **D) **Quantification showing percent of VGLUT1+ or GAD65+ puncta which also stain positive for PSD-95 or Gephyrin, respectively. N = 3 regions.

NF-κB plays an important role in regulating development of peripheral and central synaptic structures [21,38,39]. To determine whether altered synaptogenesis might underlie the improved cognitive function in IκBα^M/M ^KI mice, we examined the density of excitatory and inhibitory synaptic puncta in dissociated hippocampal neuronal cultures from WT and IκBα^M/M ^KI mice. After 14-15 DIV, cultures were fixed and stained with antibodies against the dendritic marker MAP2 and either the glutamatergic presynaptic marker VGLUT1 or the GABAergic presynaptic marker VGAT. We found a significant increase in the density of VGLUT1^+ ^puncta along MAP2^+ ^dendrites in IκBα^M/M ^KI hippocampal cultures compared to WT hippocampal cultures (Figure 4A-B, p < 0.05). Furthermore, cultures from IκBα^M/M ^KI mice had a decreased density of VGAT^+ ^puncta along MAP2^+ ^dendrites compared to WT cultures (Figure 4A-B, p < 0.001). To determine what percentage of presynaptic puncta represented bona-fide synapses, we performed immunostaining with antibodies against the presynaptic marker VGLUT1 or GAD65 and the postsynaptic marker PSD-95 or Gephyrin. We found that more than 85% of VGLUT1^+ ^puncta in our hippocampal cultures also stained positive for PSD-95 (Figure 4C-D), and over 95% of GAD65^+ ^puncta stained positive for Gephyrin (Figure 4C-D), confirming that these presynaptic puncta indeed represent bona-fide synapses. Taken together, these studies reveal an increase in excitatory and decrease in inhibitory synaptic density that suggests altered synaptic connectivity within IκBα^M/M ^KI hippocampal networks.

### Hippocampal neurons from IκBα^M/M ^KI mice exhibit spontaneous burst firing and hyperexcitability

**Figure 5:**
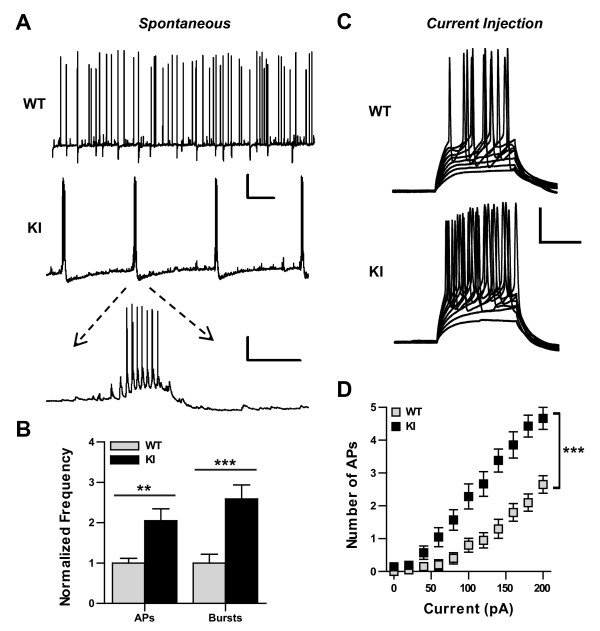
**IκBα^M/M ^KI neurons exhibit increased spontaneous burst firing and hyperexcitability in culture**. **A) **Example traces of spontaneous action potential (AP) firing from WT and KI hippocampal neurons recorded under whole cell patch in current clamp mode. Scale bar = 20 mV/10 sec (top), 20 mV/1 sec (bottom). **B) **Quantification of the frequency of spontaneous AP firing and frequency of burst firing normalized to WT levels. N = 28-32 traces, ** = p < 0.01, *** = p < 0.001. **C) **Example traces of evoked AP firing from WT and KI hippocampal neurons following a series of current injections. Scale bar = 20 mV/50 msec. **D) **Quantification of number of AP fired during indicated magnitude of current injection. N = 20-21 neurons, *** = p < 0.001.

Imbalances of excitatory and inhibitory synaptic transmission can alter the excitability of neuronal networks and contribute to epileptogenesis [40,41]. To determine if the changes in synaptic connectivity present in IκBα^M/M ^KI hippocampal neurons alters their overall excitability, we performed whole cell patch clamp recordings of cultured neurons. First, we recorded from neurons in current clamp mode to measure spontaneous action potential (AP) firing. While WT neurons typically fired single action potentials (Figure 5A, top), IκBα^M/M ^KI neurons instead regularly fired bursts of action potentials (Figure 5A, middle), which in higher detail seemed to arise from periods of depolarization followed by prolonged periods of hyperpolarization (Figure 5A, bottom). We quantified the total number of APs fired and found that this was increased in IκBα^M/M ^KI neurons (Figure 5B, p < 0.01). We also quantified the number of burst events and found that this was also significantly increased in IκBα^M/M ^KI neurons (Figure 5B, p < 0.001). Next, we measured intrinsic excitability by performing progressively increasing current injections and counting the number of APs elicited by each injection. Hippocampal neurons from IκBα^M/M ^KI mice began firing APs with weaker current injections than WT neurons and continued to fire more APs with each subsequent current step (Figure 5C-D, p < 0.001). These data suggest that the enhanced NF-κB signaling activity in IκBα^M/M ^KI mice leads to increased excitability in hippocampal neuronal networks, which may be due in part to an increase in the intrinsic excitability of individual hippocampal neurons.

### IκBα^M/M ^KI mice exhibit increased interictal epileptiform activity *in vivo*

**Figure 6:**
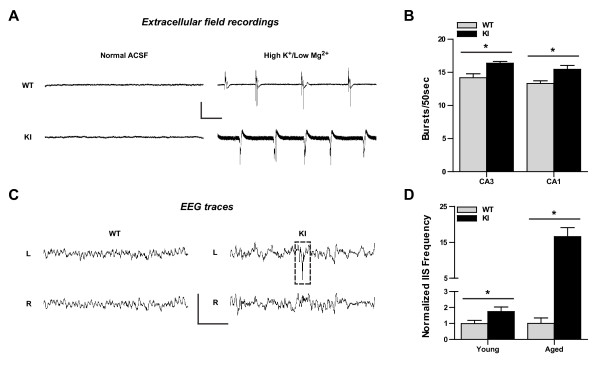
**Increased interictal epileptiform activity in IκBα^M/M ^KI mice**. **A) **Representative extracellular field recordings of acute hippocampal slices from WT or KI mice before and after perfusion with modified ACSF containing high K+/low Mg2+ to induce spontaneous interictal-type burst firing. Scale bar = 2 mV/2 sec. **B) **Quantification of number of bursts per 50 sec recording in recordings from area CA3 or CA1. N = 5-24 recordings, * = p < 0.05. **C) **Representative EEG recording from a WT mouse (left) and KI mouse (right) depicting spontaneous interictal spike (IIS) activity (dashed box) recorded chronically in awake and behaving KI mice. Scale bar = 600 μV/1 sec. **D) **Quantification of IIS frequency per hour of EEG recording normalized to WT levels in young and aged mice. N = 6-47 recordings, * = p < 0.05.

Increased excitatory synaptic connectivity and overall hyperexcitability in dissociated hippocampal neurons suggested that IκBα^M/M ^KI mice may be prone to hypersynchrony and epileptiform activity *in vivo*. To test this, we performed extracellular field recordings in acute hippocampal slices prepared from 4-5 mo old WT and IκBα^M/M ^KI mice. Under normal ACSF, we did not detect spontaneous activity in either WT or IκBα^M/M ^KI slices (Figure 6A, left). We next perfused the slices with a modified ACSF containing high K^+ ^(8.5 mM) and low Mg^2+ ^(0.5 mM), a protocol previously shown to induce interictal-type burst firing [33]. Under these conditions, we detected frequent spontaneous burst firing in hippocampal slices from both groups (Figure 6A, right). However, when we quantified the number of bursts in each recording, we found this was significantly increased in slices from IκBα^M/M ^KI mice in both area CA3 (Figure 6B, p < 0.05) and area CA1 (Figure 6B, p < 0.05), implying that hippocampal networks in these mice are more prone to generation of epileptiform activity. To determine if IκBα^M/M ^KI mice also displayed abnormal cortical hypersynchrony *in vivo*, we implanted cortical electrodes and employed prolonged video-EEG monitoring to record EEG traces and behavior from 4-5 mo old (young) WT and IκBα^M/M ^KI mice. While no electrographic or behavioral seizures were noted, we observed the presence of abnormal spontaneous epileptiform activity, appearing as sharp interictal spike (IIS) discharges in the EEG traces from IκBα^M/M ^KI mice (Figure 6C, right) compared to the relatively quiet EEG traces from WT mice (Figure 6C, left). We quantified the frequency of IIS events per hour of recording and found that this was increased in IκBα^M/M ^KI mice compared to WT mice (Figure 6D, p < 0.05). When we performed these recordings in 11-15 mo old mice (aged), we found that this increase was much more pronounced (Figure 6D, p < 0.05), implying an age-dependent progression of cortical hypersynchrony in IκBα^M/M ^KI mice. Since interictal spiking is a hallmark of epileptic brain, these results extend our *in vitro *experiments and suggest that enhanced NF-κB activity leads to increased neuronal network synchrony and activity *in vivo*, likely due to similar altered synaptic balance and hyperexcitability as demonstrated in IκBα^M/M ^KI hippocampal neuronal cultures.

## Discussion

We employed a mouse knock-in model in which we genetically disrupted the IκBα autoinhibitory loop by mutating the κB binding sites in the IκBα promoter, which abolishes NF-κB binding and promotion of IκBα expression, leading to delayed resynthesis of IκBα and enhanced NF-κB activity in multiple peripheral organs [28]. Here, we demonstrate that this IκBα autoinhibitory loop operates in the central nervous system (CNS) to tightly regulate NF-κB activity and is required to maintain normal levels of excitability. Specifically, we found that the normal NF-κB directed resynthesis of IκBα is delayed in neurons from IκBα^M/M ^KI mice. It is important to note that mutation of the κB sites does not completely abolish IκBα expression, as this would lead to an early postnatal lethality [26,27]. Rather, we see almost normal expression of IκBα at baseline in IκBα^M/M ^KI neurons. Instead, the NF-κB mediated rapid resynthesis of IκBα is blocked, with IκBα gradually reappearing after prolonged TNFα stimulation. This lagging IκBα resynthesis is possibly mediated by the activity of other transcription factors at the IκBα promoter, such as SP1, CREB, or AP-1 [25]. As a consequence, we believe that NF-κB is not constitutively active within IκBα^M/M ^KI neurons, but rather undergoes stronger and prolonged activation in response to each acute stimulation.

There are over 150 stimuli known to activate NF-κB, ranging from infectious pathogens and inflammatory cytokines to growth factors and hormones [2]. In this study, we utilized *in vitro *stimulation with TNFα and IL-1β to directly activate NF-κB in neuronal cultures. Within the CNS, these cytokines are secreted not just in response to injury, but also during normal brain function, specifically following changes in neuronal activity [42,43] or during cognitive tasks [44]. Furthermore, application of glutamate or KCl activated NF-κB in cultured neurons [13,45] indicating that activation of glutamate receptors or neuronal depolarization both can lead to NF-κB activation. As these occur spontaneously within neural networks, NF-κB activity can be detected under baseline conditions *in vitro *and *in vivo *[13,46]. Thus, we believe that IκBα^M/M ^KI mice represent a unique model of increased NF-κB activity following endogenous activation and allow the study of NF-κB under physiologic conditions.

One consequence of enhanced NF-κB activation is an improvement in both contextual and spatial learning and memory in IκBα^M/M ^KI mice. Numerous studies have been published implicating the role of NF-κB in learning and memory. Knockout of the p65 subunit of NF-κB (balanced with knockout of TNFR to bypass embryonic lethality) leads to a deficit in spatial memory formation [13], while knockout of the p50 subunit leads to an impairment in active avoidance memory [12]. Similarly, inhibition of NF-κB activation via transgenic overexpression of an IκBα super-repressor in the forebrain leads to impaired spatial memory formation [14]. A bioinformatics analysis demonstrated that many of the genes whose expression was altered by conditioned fear training were regulated by the NF-κB subunit c-Rel [15], and that c-Rel^-/- ^mice demonstrate a deficit in contextual memory formation [16]. These studies provide strong evidence that loss of NF-κB function leads to learning and memory deficits. Importantly, our studies demonstrate that the converse is also true, that boosting NF-κB signaling *in vivo *can lead to enhanced learning and memory.

In addition to improved cognitive performance, enhanced NF-κB activity also promotes neuronal hyperexcitability and the spontaneous firing of bursts of action potentials. *In vivo*, IκBα^M/M ^KI mice display elevated seizure-like activity, evidenced by increased epileptiform activity in hippocampal slices and increased interictal spike (IIS) frequency on cortical EEG. While it is not clear whether IIS events drives epileptogenesis or are an adaptive mechanism to prevent ictal events [47], IIS typically occur between seizure episodes and suggest a pro-epileptic phenotype in IκBα^M/M ^KI mice. At the neuronal level, IIS are caused by a slow depolarization current known as a paroxysmal depolarizing shift (PDS) and may reflect an increase in excitatory or a decrease in inhibitory connectivity within the network [48]. Our immunostaining experiments demonstrate that both of these changes in synaptic density are present in IκBα^M/M ^KI hippocampal neuronal cultures, and our patch-clamp recordings reveal characteristic PDS currents, suggesting that this imbalance forms the basis for IIS activity in IκBα^M/M ^KI mice. Alternatively, increased NF-κB activity may be directly enhancing the intrinsic excitability of neurons, which can in turn drive changes in synaptic connectivity [49]. This hypothesis is supported by the increased action potential firing in response to current injections in IκBα^M/M ^KI neurons, however very little is known about the role of NF-κB in regulating voltage-gated ion channels or leak channels to alter membrane excitability. In all likelihood, both synaptogenesis and intrinsic excitability are affected in IκBα^M/M ^KI mice and together contribute to the improved cognition and increased seizure-like activity.

Given its known role as a transcription factor, enhanced NF-κB activity must be exerting its effect on cognitive performance and network excitability through altered gene transcription. However, given the extremely large and diverse set of target genes thought to be regulated by NF-κB [2], the precise molecular mechanism has proven difficult to define. We measured the mRNA and protein levels of various suspected target genes which play important roles in neuronal function, including the AMPA-type glutamate receptor subunit GluR1 [50] and the NMDA-type glutamate receptor subunit NR1 [51], but were unable to find consistent changes in brain tissue or neuronal cultures. We believe this may be a consequence of the subtle nature of our genetic model, and that the phenotypes evident in IκBα^M/M ^KI mice may be a result of the cumulative effect of small changes in many genes. Additionally, gene transcription in IκBα^M/M ^KI mice may be most altered during specific contexts. Recent detailed work from the Meffert lab demonstrated that NF-κB directly regulated excitatory synaptogenesis, but only during developmental or plasticity-induced periods of rapid synapse development, and was not active during maintenance of synapses in mature neurons [21]. Interestingly, this effect was due to NF-κB-dependent transcription of the scaffolding protein PSD-95, an important regulator of excitatory synapses [52] which, through interaction with the cell adhesion molecule neuroligin, can also modulate the balance of excitatory and inhibitory synapses [41]. It will be interesting to see whether expression of PSD-95 is enhanced in IκBα^M/M ^KI mice during contextual or spatial memory consolidation.

While the focus of our experiments was on neuronal function, IκBα^M/M ^KI mice possess mutations of the IκBα promoter in all cell types. Enhanced NF-κB activity in resident glial cells might lead to increased levels of inflammatory cytokines in the brain and is thought to drive the neurotoxicity seen in chronic neurodegenerative disorders such as Alzheimer's disease or Parkinson's disease [53]. We see no evidence of neuronal loss in IκBα^M/M ^KI mice, perhaps due to a protective effect of increased NF-κB in neurons, or because the enhancement of NF-κB is too mild to trigger toxicity. Cytokine secretion has also been shown to modulate neuronal function and excitability [54,55]. Specifically, secretion of TNFα from glial cells can increase neuronal expression of AMPA-type glutamate receptors [42,43], and infusion of low levels of IL-1β into the brain can improve contextual learning and memory [44]. Furthermore, intra-hippocampal injections of IL-1β can lead to an increase in IIS frequency and sensitivity to kainate-induced seizures [56]. While we cannot exclude the possibility of contribution from these other cell types to the improved cognitive performance and hyperexcitability in IκBα^M/M ^KI mice, our experiments using primary neuronal cultures strongly suggest a direct effect in neurons.

## Conclusions

Overall, the results of our experiments confirm a role for NF-κB in regulating the formation of synaptic connections and encoding of long term memory, and demonstrate the importance of the IκBα autoinhibitory loop in properly regulating endogenous NF-κB activity in neurons to ensure healthy physiologic levels. On the one hand, we have shown that disruption of this loop can lead to enhanced learning and memory. Indeed, these results suggest that pharmacologic activation of NF-κB might be a viable therapeutic option to improve cognitive function in various forms of dementia, such as Alzheimer's disease, or following traumatic or ischemic brain injury. However as with any strategy that impairs synaptic inhibition, this must be carefully titrated so that NF-κB activity remains within a physiologic range to prevent an imbalance in synaptic strength and the potential promotion of seizure activity.

## Competing interests

The authors declare that they have no competing interests.

## Authors' contributions

DS, PC, and HZ conceived and designed the overall study with help from JN. DS, LY, JR performed the experiments and analyzed the data. DS wrote the paper with help from all co-authors. All authors read and approved the final manuscript.
